# Supplementary material for: Continental-scale analysis of shallow and deep groundwater contributions to streams
Source: Nat Commun. 2021 Mar 4;12:1450. doi: 10.1038/s41467-021-21651-0 (PMC7933412; doi:10.1038/s41467-021-21651-0)
Supplement: Supplementary file 1 — Supplementary Information [file 41467_2021_21651_MOESM1_ESM.pdf]

Supplemental Materials

Supplemental Figure 1: Amplitude Ratio and Phase Lag Histograms of Complete Dataset

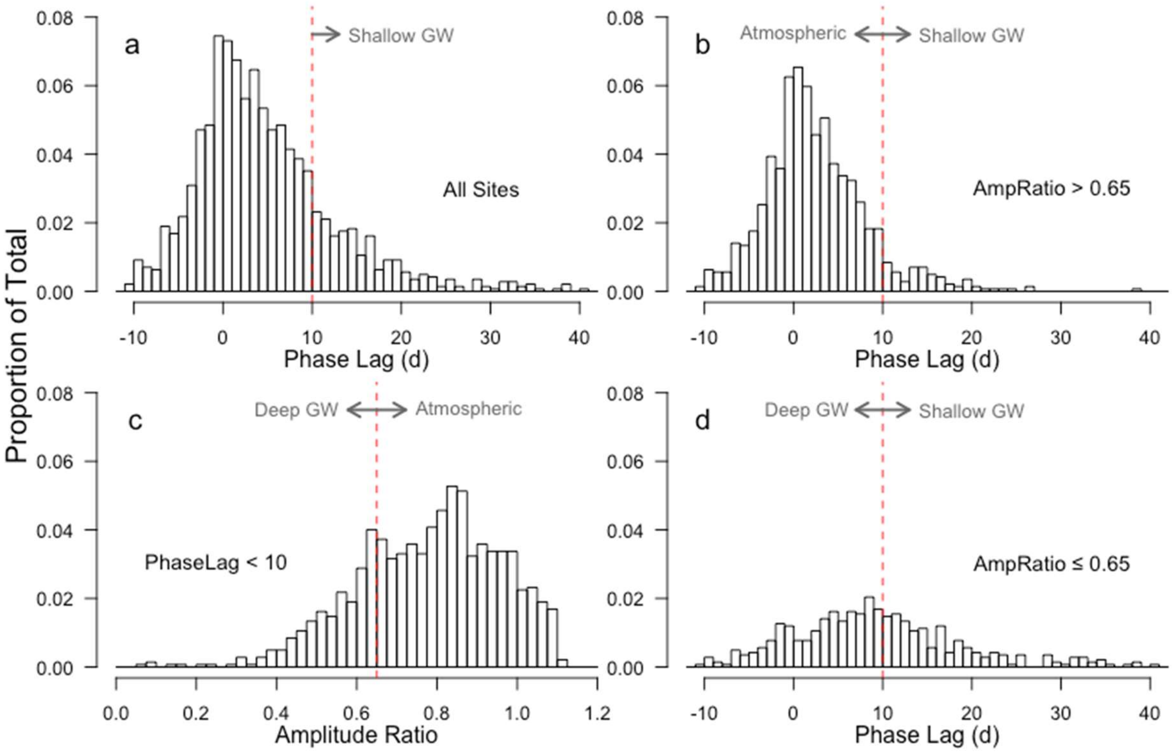

Supplemental Figure 1 depicts the data distribution and placement of thresholds of the paired air – stream water temperature annual signals. As discussed in the text, the thresholds chosen for this national scale data set for air- stream water phase lag (10d) and air – stream water amplitude ratio (0.65) are conservative and the distribution of annual signal metrics within our groundwater contribution categories indicate that the groundwater category thresholds occur near natural population breaks.

Supplementary Table 1: Physiographic Region and Province Analysis

	Continental Classifications of Groundwater Connectivity						
	Deep GW Signature		Shallow GW Signature		Atmospheric Signature		
Physiographic Regions/Provinces	Count	Percent	Count	Percent	Count	Percent	Total
APPALACHIAN HIGHLANDS	74	20%	48	13%	251	67%	373
APPALACHIAN PLATEAUS	4	7%	2	3%	54	90%	60
BLUE RIDGE	17	26%	19	29%	29	45%	65
NEW ENGLAND	40	31%	9	7%	79	62%	128
PIEDMONT	2	3%	5	7%	67	91%	74
VALLEY AND RIDGE	11	24%	13	28%	22	48%	46
ATLANTIC PLAIN	5	3%	11	6%	166	91%	182
COASTAL PLAIN	5	3%	11	6%	166	91%	182
INTERIOR HIGHLANDS	2	13%	3	19%	11	69%	16
OUACHITA		0%		0%	5	100%	5
OZARK PLATEAUS	2	18%	3	27%	6	55%	11
INTERIOR PLAINS	25	15%	16	10%	126	75%	167
CENTRAL LOWLAND	18	15%	6	5%	94	80%	118
GREAT PLAINS	7	21%	7	21%	20	59%	34
INTERIOR LOW PLATEAUS		0%	3	20%	12	80%	15
INTERMONTANE PLATEAUS	29	25%	18	16%	68	59%	115
BASIN AND RANGE	5	17%	1	3%	24	80%	30
COLORADO PLATEAUS	10	27%	2	5%	25	68%	37
COLUMBIA PLATEAU	14	29%	15	31%	19	40%	48
LAURENTIAN UPLAND	4	27%	1	7%	10	67%	15
SUPERIOR UPLAND	4	27%	1	7%	10	67%	15
PACIFIC MOUNTAIN SYSTEM	83	28%	81	27%	133	45%	297
CASCADE-SIERRA MOUNTAINS	40	32%	48	38%	38	30%	126
PACIFIC BORDER	43	25%	33	19%	95	56%	171
ROCKY MOUNTAIN SYSTEM	42	16%	114	44%	103	40%	259
MIDDLE ROCKY MOUNTAINS	8	31%	11	42%	7	27%	26
NORTHERN ROCKY MOUNTAINS	16	14%	83	74%	13	12%	112
SOUTHERN ROCKY MOUNTAINS	18	16%	18	16%	76	68%	112
WYOMING BASIN		0%	2	22%	7	78%	9
Total	264	19%	292	21%	868	61%	1424

Supplemental Table 1 includes the count values and percentages of total shallow groundwater, deep groundwater, and atmospheric signatures within each United States physiographic region and physiographic province, which are categorizations based on large scale geomorphology.
